# Aggregated Alpha-Synuclein Transfer Efficiently between Cultured Human Neuron-Like Cells and Localize to Lysosomes

**DOI:** 10.1371/journal.pone.0168700

**Published:** 2016-12-28

**Authors:** Jakob Domert, Christopher Sackmann, Emelie Severinsson, Lotta Agholme, Joakim Bergström, Martin Ingelsson, Martin Hallbeck

**Affiliations:** 1 Department of Clinical Pathology and Department of Clinical and Experimental Medicine, Linköping University, Linköping, Sweden; 2 Department of Public Health/Geriatrics, Rudbeck Laboratory, Uppsala University,Uppsala,Sweden; Louisiana State University Health Sciences Center, UNITED STATES

## Abstract

Parkinson’s disease and other alpha-synucleinopathies are progressive neurodegenerative diseases characterized by aggregates of misfolded alpha-synuclein spreading throughout the brain. Recent evidence suggests that the pathological progression is likely due to neuron-to-neuron transfer of these aggregates between neuroanatomically connected areas of the brain. As the impact of this pathological spreading mechanism is currently debated, we aimed to investigate the transfer and subcellular location of alpha-synuclein species in a novel 3D co-culture human cell model based on highly differentiated SH-SY5Y cells. Fluorescently-labeled monomeric, oligomeric and fibrillar species of alpha-synuclein were introduced into a donor cell population and co-cultured with an EGFP-expressing acceptor-cell population of differentiated neuron-like cells. Subsequent transfer and colocalization of the different species were determined with confocal microscopy. We could confirm cell-to-cell transfer of all three alpha-synuclein species investigated. Interestingly the level of transferred oligomers and fibrils and oligomers were significantly higher than monomers, which could affect the probability of seeding and pathology in the recipient cells. Most alpha-synuclein colocalized with the lysosomal/endosomal system, both pre- and postsynaptically, suggesting its importance in the processing and spreading of alpha-synuclein.

## Background

Parkinson’s disease (PD) is the second most common neurodegenerative disorder and is neuropathologically characterized by Lewy bodies and Lewy neurites: intracellular accumulations of alpha-synuclein (α-syn). Other α-synucleinopathies include dementia with Lewy bodies (DLB), multiple system atrophy (MSA) and the Lewy body variant of Alzheimer’s disease (AD). Depending on the distribution of lesions, these disorders follow a chronic and progressive course with various degrees of motor, cognitive, behavioral and autonomic symptoms. In PD, the early brain pathology can typically be identified in the substantia nigra, followed by involvement of other areas of the midbrain and limbic system and then engagement of the neocortex at advanced disease stages (reviewed in [1]). Thus, the PD brain presents a hierarchical neuroanatomical picture in which distinct neuronal and glial cell types are affected in a pattern corresponding to axonal projections [2, 3], suggesting that the disease may progress via the transfer of pathological proteins between interconnected brain regions.

An indication that α-syn may propagate disease by spreading from cell to cell comes from the *post mortem* evaluation of PD brains with transplanted fetal neurons. These brains displayed α-syn inclusions within the grafted tissues which presumably transferred from the host tissue [4, 5]. Studies on animal models have demonstrated that inoculation of preformed α-syn fibrils or of brain homogenate from PD patients or α-syn transgenic mice was sufficient to cause all the major pathological changes observed in PD, including protein aggregation, neurodegeneration and neuroinflammation [6–8]. More recently, experimental evidence from rat models suggests that α-syn may enter the brain via the gut [9], this finding is interesting given that peripheral neurons in the colon mucosa of PD patients also display α-syn pathology during the early stages of the disease process [10]. Taken together, the observations in animal models and PD patients suggest a prion-like mechanism for the propagation of α-syn pathology. This process would include the formation of aggregates and the failure to degrade such pathogenic species in the initial cell, followed by subsequent uptake of the secreted proteins by a recipient cell. As a result, axonal degeneration and neuronal death may occur both in cells releasing these toxic aggregates and in cells receiving them.

Alpha-synuclein undergoes a stepwise transformation from its monomeric form to fibrils via a number of intermediately sized soluble aggregates (reviewed in [11]). A growing body of evidence indicates that pre-fibrillar species, such as oligomers, may have particularly cytotoxic effects, whereas the fibrils themselves may represent a neuroprotective response [12–14]. Extracellular species of both oligomeric and fibrillar α-syn can be internalized and induce intracellular seeding aggregation in cultured cells [6, 15, 16]. Such effects can be potentiated by failure in the proteasomal [17, 18] or lysosomal [19] degradation systems or by reduced levels/activity of intra- and extracellular proteases [20]. Despite the increased knowledge in this field, our understanding of the roles of different α-syn aggregates for the propagation of pathology in Lewy body disorders remains incomplete. However, it has been shown that α-syn aggregates can cause cellular toxicity depending on intracellular locations [21], and evidence suggests detrimental effects on both mitochondrial [22] and lysosomal [19] functions, as well as autophagy [23] and calcium homoeostasis [24].

To elucidate the different steps in the propagation process and investigate how the cell-to-cell transfer of pathological proteins can be prevented or halted, appropriate cell models are needed. We have established a model based on human cells that are differentiated to have neuron-like phenotypes and properties [25]. With this model, we have previously shown that oligomeric amyloid-β (Aβ) can transfer across the synapse and confer cellular toxicity post-synaptically [26]. In the current study, we used the same cell model to examine the uptake and transfer of different species of α-syn. We also investigated the subcellular localization of such species in order to further understand their modes of action and how they are processed within the recipient cells.

## Material and Methods

### Preparation of monomeric, oligomeric and fibrillar Cy3 conjugated α-synuclein

Production of recombinant α-syn monomers and generation of oligomers and fibrils, respectively, was performed as previously described [27]. In short, to generate oligomeric α-syn, the aldehyde 4-hydroxy-2-nonenal (HNE, Cayman Chemicals) was added to monomeric α-syn (140 μM, equilibrated in 50 mM sodium phosphate buffer, pH 7.4) with a concentration corresponding to a molecular ratio of 30:1 (HNE: α-syn) and incubated at 37°C for 18 h. Unbound HNE was removed by using Zeba desalting spin column according to the manufacturer’s instructions (Thermofisher). To generate fibrillar α-syn, unmodified monomeric protein (140 μM) equilibrated in 50 mM sodium phosphate buffer, 150 mM NaCl, pH 7.4 was incubated for 4–5 d at 37°C on a polypropylene 96-well plate (Greiner Bio ONE, Frickenhausen, Germany), which was agitated at 900 rpm on a Ika MS3 basic shaker (Ika, Staufen, Germany). The oligomers have been shown to have a beta-sheet rich structure with a protofibrillar morphology with a molecular weight of 2000 kDa. Since they do not aggregate to form fibrils they are defined as oligomers. The fibrils are typical non-branching amyloid-like fibrils about 1 μm in length [27]. Conjugation of monomeric, oligomeric or fibrillar α-syn was carried out using the Cy3 monoreactive dye pack labeling kit (GE Healthcare) per the manufacturer’s protocol. The concentration of the various alpha-synuclein species given in subsequent experiments refers to the starting concentration of monomeric protein. The species were characterized using standard uranyl-acetate staining procedures for transmission electron microscopy.

### Fluorescence intensity of α-syn species

Cy3 labeled monomers, oligomers and fibrils were diluted in PBS to concentrations of 4 μM, 2 μM, 1 μM, 0.5 μM, 0.25 μM and 0.125 μM. The dilutions were added to a black bottomed 96-well plate then measured in a Victor^3^ plate reader (PerkinElmer, Inc.). The samples were excited at 550 nm and emission measured at 572 nm.

### Cell culture, differentiation and co-culture of SH-SY5Y cells

Differentiation and co-culture were performed as previously described [25, 26]. In brief, the SH-SY5Y cells (ECACC: Sigma-Aldrich) were cultured and pre-differentiated for 7 d using 10 μM retinoic acid (RA; Sigma-Aldrich).

For the co-culture of acceptor cells, pre-differentiated cells were mixed with extracellular matrix (ECM) gel (BD Bioscience) at a 3:1 ratio in 4-well Lab-Tek chambers (Thermo Scientific Nunc). The cells were further differentiated with MEM supplemented with brain-derived neurotrophic factor (BDNF, 50 ng/ml, PeproTech), neuregulin β1 (NRGβ1, 10 ng/ml, R&D Systems), nerve growth factor (NGF, 10 ng/ml, R&D Systems) and vitamin D3 (VitD3, 24 nM, Sigma-Aldrich). After 10 d of differentiation, the cells were transfected with BacMam 2.0 early endosomes Rab5a-GFP (Life Technologies) at a final concentration of 15 particles per cell, yielding fluorescently labeled acceptor cells distinguishable from the donor cell population. No changes to cellular morphology, behavior (including transfer) or viability was seen in this or our previous studies using this labeling technique [26, 28].

In parallel, donor cells were incubated in MEM with 1 μM of Cy3-labeled monomeric, oligomeric or fibrillar α-syn for 3 h at 37°C to allow cells to take up the proteins. Cells were then washed, trypsinized and reseeded in ECM gel on top of the acceptor cells and co-cultured in RA supplemented MEM for 24 h or 48 h at 37°C [26]. Subsequently, cells were fixed and mounted with ProLong Gold antifade reagent with DAPI (Life Technologies). To evaluate α-syn uptake, donor cells were treated with α-syn as described above but reseeded into 24-well plates on 12 mm glass slides (VWR International). All other procedures were the same as for the donor cells in the co-culture.

### Immunocytochemistry

Donor cells were permeabilized with 0.1% saponin and 5% FBS for 20 min. After fixation, co-cultures were washed with 100 mM glycine in PBS and permeabilized/blocked in a PBS buffer containing 0.02% Triton X-100, 0.1% BSA, and 0.05% Tween-20, along with 10% goat serum (Sigma-Aldrich) and 1% goat anti-mouse IgG (Dako). The following primary antibodies were used: mouse anti-KDEL (marker for endoplasmic reticulum and cis-Golgi network, 1:400; Enzo Life Sciences), mouse anti-SV2 (Synaptic vesicle glycoprotein 2a, marker for the synapse and synaptic vesicles, 1:500; deposited by K.M. Buckley to the Developmental Studies Hybridoma Bank, The University of Iowa), mouse anti-TSG101 (tumor susceptibility gene 101, marker for multi-vesicular bodies (MSBs) and other endosomal compartments, 1:400; Thermo Pierce), mouse anti-VAMP2/synaptobrevin (Vesicle-associated membrane protein 2, marker for the synapse and synaptic vesicles, 1:100; Synaptic Systems), and mouse anti-LAMP2 (Lysosomal associated membrane protein 2, marker for lysosomes/endosomes, 1:100; Southern Biotechnology). Incubation with the indicated primary antibody (overnight, 4°C) was followed by incubation with a secondary antibody conjugated with either Alexa Fluor 405 or Alexa Fluor 488 (Invitrogen) (75 min for donor cells only or 45 min for co-culture, RT). Finally, slides were mounted with ProLong containing DAPI.

### Confocal microscopy

Images were acquired with a Zeiss LSM 700 inverted confocal microscope, using a 63X/1.40 oil objective. The included images are representative of the findings, and at least five photos were captured for each time point and marker for each experiment. A minimum of three experiments were collected for each immunocytochemical marker.

### Image analysis of uptake, transfer and cellular localization of α-syn

Zen 2011 (Carl Zeiss) and Volocity 3D version 6.0 (Perkin Elmer) software were used for all image analysis performed. The proportions of donor cells that had taken up Cy3-labeled α-syn (monomers, oligomers or fibrils) at 24 h or 48 h post-treatment were assessed based on confocal images by three investigators who were blinded to the nature of the samples. An average of 1855 donor cells from an average of 67 pictures per treatment and time point were counted. Each cell was judged as containing labeled α-syn or not, regardless of the amount. Similarly, the proportions of acceptor cells containing transferred α-syn monomers, oligomers or fibrils after 24 h of co-culture were quantified on confocal images by three investigators who were blinded to the nature of the samples. An average of 460 acceptor cells from an average of 83 pictures per treatment were counted. The results were expressed as binominal data and analyzed as frequency tables. Significance levels were determined through Chi-square tests followed by Bonferroni corrections.

The intracellular localization of the labeled α-syn was quantified from confocal images with either donor cells (24 h after treatment) or acceptor cells in co-cultures (after 24 h). Regions of interest were manually mapped over each included cell soma (donor or acceptor cells). Within these regions, colocalization of α-syn to the respective marker for the intracellular compartments was measured using Volocity software and calculated as the Manders colocalization coefficient (M1), describing the proportion of the labeled α-syn within the cell that was co-localized with the target structure. Data are expressed as the mean ± SEM, and statistical comparisons were made using one-way ANOVA followed by the Bonferroni correction (GraphPad Prism) with a significance level of *p* < 0.05.

## Results

### Uptake and toxicity of α-syn in differentiated SH-SY5Y cells

**Fig 1:**
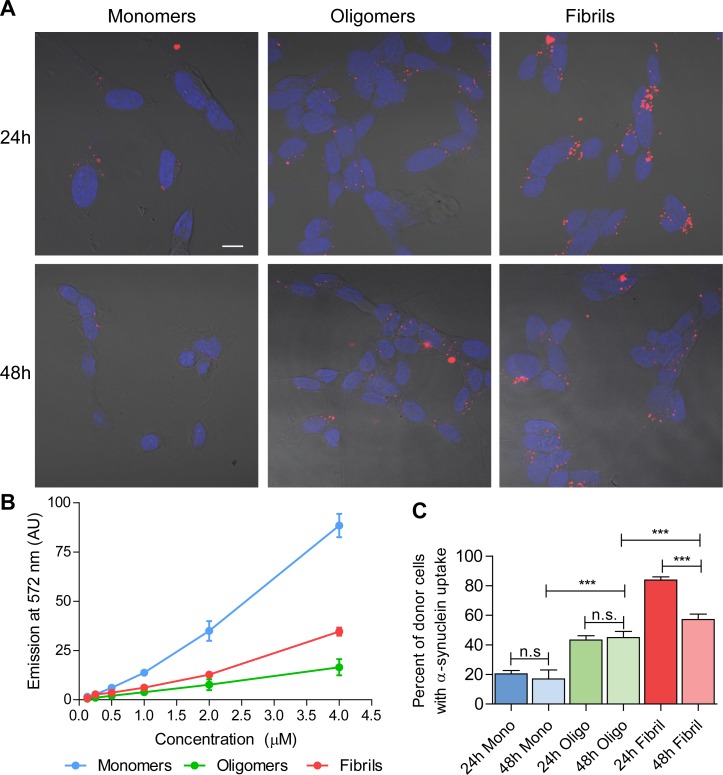
Uptake of α-synuclein in differentiated SH-SY5Y donor cells. SH-SY5Y cells differentiated for 7 d with 10 μM retinoic acid were treated for 3 h with 1 μM monomeric, oligomeric or fibrillar Cy3-labeled α-synuclein and reseeded onto cover slips. After 24 h or 48 h of incubation, the cells were fixed and mounted onto slides with ProLong antifade reagent containing DAPI. (A) Confocal images showing cellular uptake of monomeric, oligomeric and fibrillar species of α-synuclein (red) and DAPI (blue) staining for the nuclei. (B) The Cy3 fluorescence intensity at different concentrations of α-syn species. (C) Quantification of α-syn uptake. Data are presented from an average of 1855 counted donor cells from an average of 67 pictures per treatment and time point. The results are presented as the mean percentage of positive transfer ± SEM. n.s. not significant, *** p<0.001, Chi-test with Bonferroni correction (Scale bar = 10 μm).

To investigate whether the different α-syn species are taken up by neuron-like SH-SY5Y cells, they were incubated with electron microscopy characterized (S1 Fig) 1 μM Cy3-fluorescently labeled monomers, oligomers or fibrils of α-syn for 3 h. Subsequently, extracellular α-syn was removed and cells were washed, trypsinized (to remove cell surface bound α-syn), then reseeded and cultured for 24 or 48 h followed by fixation for further analysis. Using confocal microscopy, the uptake of fluorescently labeled monomers, oligomers and fibrils was visible after 24 and 48 h post α-syn treatment (Fig 1A). The fluorescence intensity was investigated for the different α-syn-Cy3 species. The monomers had the strongest intensity, while oligomers and fibrils had similar intensities at most concentrations, but deviated at 4 μM, where fibrils showed stronger intensity (Fig 1B). The Cy3 labeling was performed after oligomerization and fibrillization, but from mapping of surface exposed epitopes [27] differences in the number of available epitopes for the dye to react with is unlikely to explain these differences in fluorescence intensity. Because of the differences in fluorescence intensity it was deemed impossible to accurately compare the intra-cellular labeling intensity between species, rather the number of cells containing the respective α-syn species were compared.

The proportion of cells containing labeled α-syn 24 h after treatment was highest for fibrils (83.2%), with significantly lower proportions of positive cells for oligomers (46.5%) and even lower proportions for monomers (19.7%) (Fig 1C). After 48 h, these differences between species remained. However, while the proportions of cells containing monomers and oligomers (17.0 and 46.6%, respectively) did not change significantly there was a significant decrease in the proportion of fibril-positive cells (to 60.4%) after 48 h. This reduction might be the result of quenching due to structural changes in the fibrils, or increased release to the extracellular space. As the fluorescence intensity (Fig 1B) was highest for monomers the differences in uptake and retention of different α-syn species might be higher than the numbers presented here.

Two viability assays were performed to determine whether neurotoxicity occurs in response to monomeric, oligomeric or fibrillar α-syn after 24 h. Only the monomeric species showed a small, but significant, decrease in cell viability using standard XTT assay (S2A Fig). Additionally, tubulin beading, an early marker of neurotoxicity, was examined as previously described [26]. However, none of the α-syn species showed any indication of tubulin beading seven days after α-syn treatment (S2B Fig).

### Cell-to-cell transfer of α-syn between co-cultured human cells

**Fig 2:**
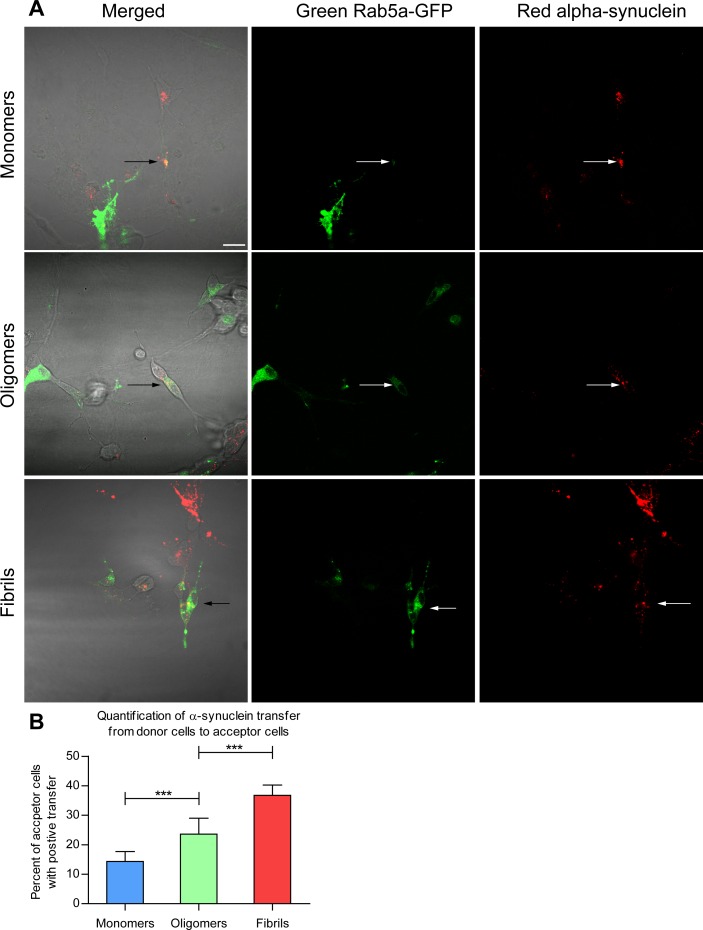
Transfer of Cy3-labeled α-syn between neuronal cells. Co-culture of neuronally differentiated donor cells containing Cy3-labeled α-syn (red) and acceptor cells transfected with EGFP-tagged endosomal Rab5a (green). (A) Confocal images show transfer (black and white arrows) of monomeric, oligomeric and fibrillar α-syn from donor cells to acceptor cells after 24 h of co-culture. (B) Quantification of acceptor cells with successfully transferred α-syn isoforms. Data are presented from an average of 460 counted acceptor cells from an average of 83 pictures per treatment. The results are presented as the mean percentage of positive transfer ± SEM. *** p<0.001, Chi-test with Bonferroni correction (Scale bar = 20 μm).

To investigate whether α-syn can be transferred between neuron-like cells, we used our recently developed donor-acceptor co-culture model, in which two subpopulations of cells form an extensive branched network with synapse-like connections, as previously described [26]. Donor cells treated for 3 h with 1 μM Cy3-labeled monomeric, oligomeric or fibrillar α-syn were reseeded on top of differentiated acceptor cells expressing a green EGFP-tagged endosomal marker (Rab5a). After 24 h, the co-culture was fixed, and the results were visualized using confocal microscopy. While the transfer from donor to acceptor cells was observed for all α-syn species (Fig 2A), the net quantity of transfer-positive acceptor cells differed significantly depending on the α-syn species used (Fig 2B). Thus, the proportion of acceptor cells that contained transferred Cy3-labeled α-syn was highest for fibrils (36.7%), followed by oligomers (23.6%) and then monomers (14.3%). In addition, the release of different species from the cultured donor cells was examined. Donor cells were feed with α-syn species as above and the supernatant was collected after 24 h. Extracellular Cy3 fluorescence was significantly increased in fibril treated cells relative to oligomer and monomer treated cells (S3 Fig).

### Both pre-synaptic and post-synaptic α-syn preferentially localized to endosome- and lysosome-related organelles

**Fig 3:**
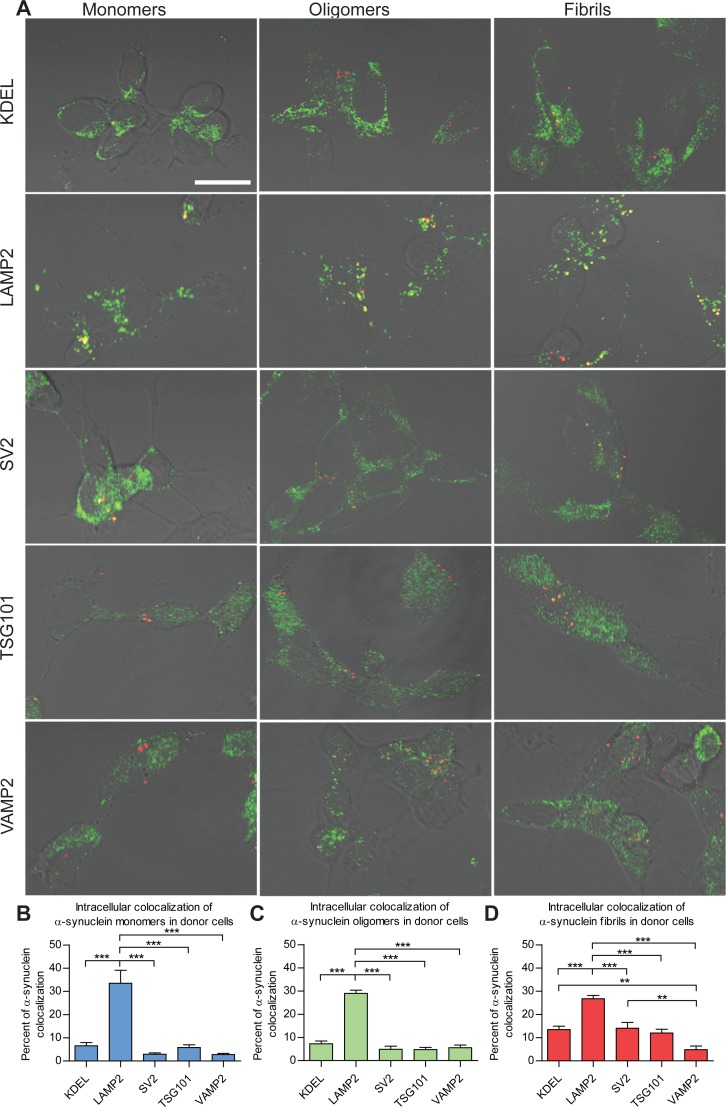
Colocalization of α-syn species and selected intracellular targets in donor cells before transfer. Monomeric, oligomeric or fibrillar Cy3-labeled (red) α-syn was added to RA-differentiated SH-SY5Y cells and incubated for 3 h. After 24 h, the cells were fixed and then subjected to immunocytochemistry using Alexa Fluor 488-conjugated secondary antibody (green) showing subcellular targets. (A) Confocal microscopy images showing α-syn (red) colocalization (yellow) with LAMP2, VAMP2, KDEL, SV2 or TSG101 (green). Graphs show quantification of the total proportions of the intracellular α-syn monomers (B), oligomers (C), and fibrils (D) that are colocalized with the respective subcellular targets. The results are presented as the mean percentage of colocalization ± SEM. ** p<0.01, *** p<0.001, one-way ANOVA with Bonferroni correction. (Scale bar = 10 μm).

**Fig 4:**
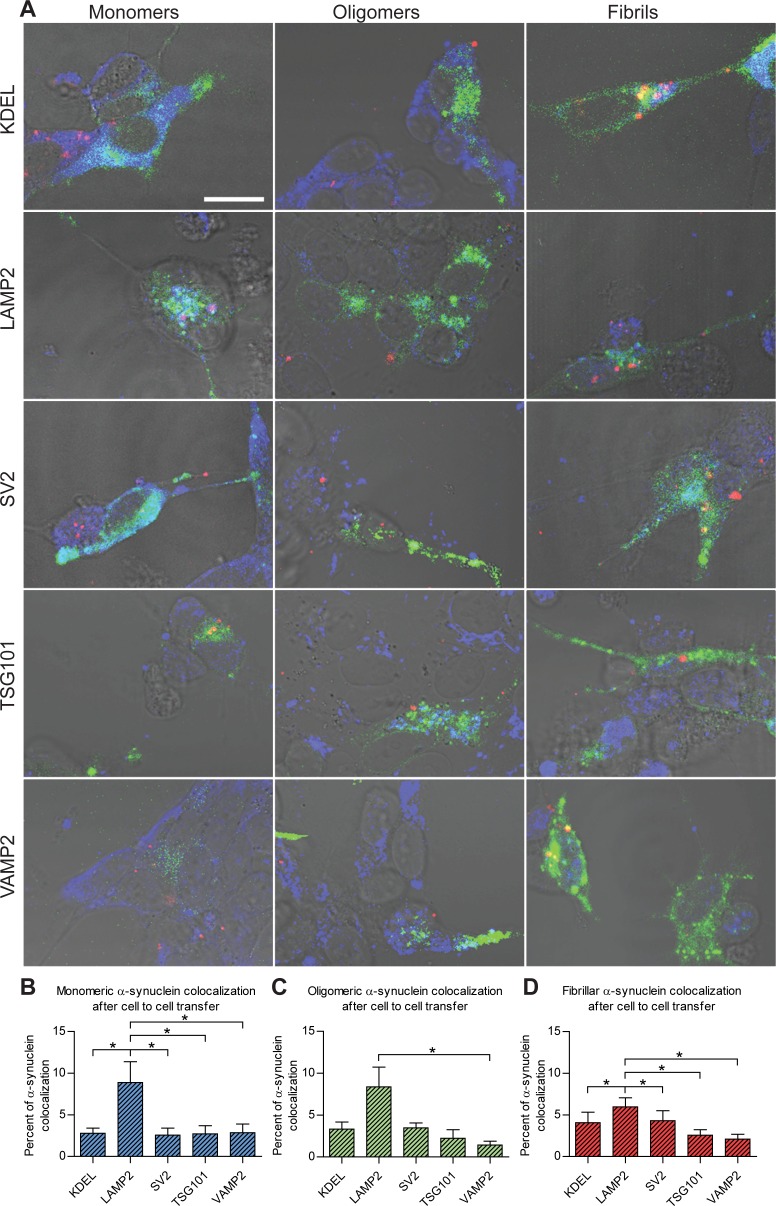
Colocalization of α-syn species and intracellular selected targets in acceptor cells after transfer. (A) Confocal images of donor and acceptor cells after 24 h co-culture show colocalization (magenta) of Cy3-labeled α-syn (red) with LAMP2, VAMP2, KDEL, SV2 or TSG101 using the Alexa Fluor 405-conjugated secondary antibody (blue) in GFP-expressing acceptor cells (green). (B-D) Quantification of the total proportions of the intracellular α-syn monomers (B), oligomers (C), and fibrils (D) that are colocalized with the respective subcellular targets. The results are presented as the mean percentage of colocalization ± SEM. * p<0.05, one-way ANOVA with Bonferroni correction. (Scale bar = 10 μm).

Using the same models as above, the sub-cellular localization of monomeric, oligomeric and fibrillar species of α-syn in pre-synaptic (donor) (Fig 3) and post-synaptic (acceptor) (Fig 4) cells was investigated 24 h after the start of the co-culture. The majority (33.5%) of pre-synaptic monomeric α-syn was found to be contained within LAMP2-positive lysosomal/endosomal vesicles (Fig 3B). In addition, α-syn monomers showed a minor association with the KDEL-positive endoplasmic reticulum (ER)-derived vesicles (6.5%) and TSG101-positive multi-vesicular bodies (MVBs, 5.8%). Oligomeric species of α-syn (Fig 3C) and fibrillar (Fig 3D) in pre-synaptic cells also colocalized primarily with organelles labeled with markers of the lysosomal/endosomal pathway (29.0% and 26.8%, respectively). Similar to monomers, minor fractions of oligomers and fibrils were also found in the KDEL-positive ER structures (7.3% and 13.5%, respectively) and TSG101-positive MVBs (4.8% and 12.0%). Furthermore, both oligomers and fibrils colocalized to a lesser degree with SV2-positive synaptic vesicles (4.9% and 14.0%) and with VAMP2-positive synaptic vesicles (5.6% and 4.9%).

Like the pattern observed for pre-synaptic donor cells, all species of α-syn were mainly found in LAMP2-positive structures within the post-synaptic acceptor cells (Fig 4A). The percentage of the transferred α-syn in the post-synaptic acceptor cells that localized to LAMP2-positive lysosomes corresponded to 8.9% for monomers (Fig 4B), 8.4% for oligomers (Fig 4C) and 6.0% for fibrils (Fig 4D). The different species of α-syn could also be found in small amounts in the other analyzed compartments in the post-synaptic (acceptor) cells, but to a lesser degree than in the pre-synaptic (donor cells).

## Discussion

In the present study, we demonstrate that monomers, oligomers and fibrils of α-syn can transfer between cells in our previously established 3D-matrix differentiated human neuroblastoma model. Culturing cells in 3D has many advantages, such as increased survival, differentiation and translates better to *in vivo* environment compared to 2D (reviewed in [29]). Using this differentiation system, the cells developed a neuron-like phenotype and express mature neuron-specific characteristics [25].

Oligomeric and fibrillar species of α-syn were detected more frequently in post-synaptic acceptor cells than were monomers. This might suggest that these species are more readily transferred and/or have higher resistance to clearance or degradation, as we have previously shown with beta amyloid isoforms [30]. Although it cannot be ruled out that some of the oligomeric and fibrillar species could partially dissociate into smaller molecular units our previous studies support the stability of the aggregates [27] and their labeling [31], and that such species could also be transferred. Moreover, our data indicate that this transfer is mainly mediated by endocytosis/exocytosis as the largest fraction of transferred α-syn is localized to the lysosomal compartment in both the pre- and post-synaptic cells at 24 h. Although α-syn oligomers have been identified as the most neurotoxic species [12–14, 19, 22–24], in the current settings with short experimental times only a small toxic effect of monomeric α-syn was seen at 24 h while neither oligomers nor fibrils showed toxicity at 24 h (XTT assay) or at 7 d (tubulin beading) indicating that, in this system, α-syn toxicity is negligible, if not absent altogether. This supports our previous studies [30] that transfer of aggregates of neurodegenerative proteins is not a result of toxicity or secondary to cell toxicity.

Our findings agree with previous reports showing localization of α-syn to synaptophysin/synaptobrevin-2 [32, 33], mitochondria [34], ER [35], MVBs and lysosomes [36]. While it is plausible that α-syn could be found in any of these compartments, we show that the majority of α-syn is localized to lysosomes or endosomal structures and that only a small fraction of intracellular α-syn is present in the other compartments investigated. Additionally, previous studies have investigated this topic using transgenic animals expressing mutant α-syn, or have utilized less differentiated cell lines, in addition to studying only a single species of α-syn. Previous studies have not investigated pre- and post-synaptic subcellular localization, being analogous to only the effects of uptake of extracellular α-syn. Considering that Lewy bodies and Lewy neurites are primarily intracellular inclusions, focus should be weighted toward the post-synaptic mediators of pathology. Here, we have investigated three different species of α-syn (monomers, oligomers and fibrils) and performed a careful examination of the subcellular localization of these species before and after cell-to-cell transfer.

The data presented here suggest that intercellular transfer of different species of α-syn primarily utilize the endocytic/exocytic transfer cycle. Both oligomeric and fibrillar α-syn were mainly localized to lysosomal and endosomal (LAMP2-positive) structures. In contrast to previous reports [32, 33], we found less α-syn localized to synaptic vesicles (SV2) and SNARE (Soluble NSF Attachment Protein Receptor)-related vesicles (VAMP2-positive). It is conceivable that a higher proportion of the endogenous α-syn would colocalize with markers of these structures because functional α-syn is involved in vesicle trafficking and SNARE vesicle fusion. In addition, the high amount of exogenously added α-syn could interfere with the normal function and localization in these cells. For example, oligomeric α-syn can inhibit SNARE membrane fusion with the pre-synaptic membrane of dopaminergic neurons [32, 37].

The present study supports the idea that the cell-to-cell spread of aggregated species of α-syn implicates several specific cellular compartments. The high degree of colocalization observed between α-syn and markers for endosomes, ER and MVBs supports the hypothesis that the endocytic process is centrally involved in the transfer process. This is consistent with findings from studies of other neurodegenerative proteins [26, 38]. A growing body of evidence points toward the importance of protein endocytic/exocytic flux in the process of neurodegenerative diseases [39–41] related not only to α-syn but also to Aβ and tau.

All tested species of α-syn were found to transfer between cells. However, the net number of affected cells after 24 h varied greatly depending on the α-syn species, with a significantly smaller proportion of acceptor cells containing monomers than oligomers, which in turn was significantly lower than for fibrils. The difference in levels between α-syn species may be related to differences in the transfer process or to the cellular ability to process the added α-syn, but most likely both mechanisms are at play. This is supported by the current finding that more fibrils were released from cells as compared to either monomers or oligomers. In addition, all α-syn species were localized mainly to compartments related to the lysosomal system. In line with previous studies [42], this supports that the differences in the amounts of internalized α-syn may be further enhanced by differences in the ability of the cells to degrade each protein species. The proteasomal and lysosomal systems have previously been linked to the processing of neurodegenerative protein aggregates [28, 30, 43, 44]. Not only are the aggregates more difficult to degrade, they can also interfere with the function of the proteasomal [17, 18] and lysosomal [19] degradation systems. Differences in degradation might also depend on conformational specificity in degradation enzymes [45].

Failure to degrade intracellular accumulations of α-syn, as well as Aβ, following proteasomal or autophagic failure, then leads to increased secretion of aggregates [30, 43, 46]. We have recently shown, using the same co-culture model, that accumulation of Aβ oligomers occurs in lysosomes and that reduced degradation within these structures leads to an increase in neuronal transfer of Aβ oligomers [30]. Given the similarities between the results of these studies, the transfer of either α-syn or Aβ appears to utilize the same cellular compartments, suggesting that the mechanisms of transfer and thus disease propagation may be related among neurodegenerative diseases. Due to these similarities, understanding the mechanism by which neurons begin to accumulate misfolded/aggregated proteins is imperative, and it may perhaps be explained by a combination between increased production of protein and decreased degradation. The lysosomal system, as a cellular protection mechanism, can digest and release partially degraded proteins via exocytosis, allowing connected and proximal cells to take up the released proteins. Assuming that the recipient cells are otherwise healthy, they might be able to degrade the aggregates; however, in the presence of cellular stress and other contributing factors, they may begin to accumulate the misfolded, aggregated proteins.

Mutations in genes related to the endosomal-lysosomal system, such as impairment of the retromer system, which is involved in endosomal trafficking, have been associated with the development of late-onset PD [47, 48]. The precise pathophysiological consequences of these mutations are still being elucidated, but studies suggest that their effects may be related to defective delivery of Cathepsin D to the endosomal-lysosomal system or to reduced formation and function of autophagosomes [49]. There is also a link between PD and Gaucher disease, which is a lysosomal storage disorder resulting from impaired activity of the lysosomal hydrolase β-glucocerebrosidase, which has also been implicated in PD [50]. This could lead to impaired processing of α syn, formation of α syn aggregates and decreased clearance of aggregates, further culminating in increased levels of neurotoxic α-syn aggregates [51].

### Conclusions

The propagation of misfolded and aggregated proteins, such as α-syn in Parkinson’s disease and other synucleinopathies, is believed to cause the progressive spread of neurodegenerative diseases. Here, we demonstrate that monomers, oligomers and fibrils of α-syn can be taken up and transferred from cell-to-cell in a 3D human neuron-like cell culture model. Oligomeric and fibrillar species of α-syn were both taken up and transferred to a greater extent than monomers, a process that seems to depend upon the endosomal/lysosomal system. Such intercellular spreading of presumably pathogenic species of α-syn may explain the propagation of disease progression in the PD brain and offer novel targets for pharmaceutical intervention.

## Supporting Information

S1 Fig **Electron microscopy characterization of labeled α-syn species.** Standard transmission electron microscopy (TEM) images show the different, and typical, characteristics of Cy3 labeled α-syn monomers (A), HNE oligomers (B) and fibrils (C). Scale bars = 500 nm.
(PDF)

S2 Fig Toxicity of alpha-synuclein. (A) XTT assay was performed on donor cells according to the manufacturer’s instructions 24h after incubation with each Cy3 labeled α-syn species. Only monomeric alpha-synuclein showed a significant decrease in cell viability after 24h. n = 3. Data are presented as mean ± SEM, ANOVA with Bonferroni’s correction. (B) As a further measure of early cellular toxicity, beading of GFP labeled tubulin was examined as described previously [1,2]. 7 days after addition of 1μM Cy3 labeled α-syn (red), no indication of tubulin beading could be seen in response to any of the α-syn species. Blue = DAPI. Images are representative, n = 3.
(PDF)

S3 Fig Extracellular secretion from donor cells. The supernatant from donor cells was collected 24h after the same uptake conditions as in all other experiments. Extracellular Cy3 fluorescence was significantly increased in fibril treated cells relative to oligomer and monomer treated cells supporting an increased release of fibrils versus monomers or oligomers. n = 3 for each group. Data are presented as mean ± SEM, ANOVA with Bonferroni’s correction.
(PDF)
