# Supplementary material for: Aggregated Alpha-Synuclein Transfer Efficiently between Cultured Human Neuron-Like Cells and Localize to Lysosomes
Source: PLoS One. 2016 Dec 28;11(12):e0168700. doi: 10.1371/journal.pone.0168700 (PMC5193351; doi:10.1371/journal.pone.0168700)
Supplement: S1 Fig — Electron microscopy characterization of labeled α-syn species. Standard transmission electron microscopy (TEM) images show the different, and typical, characteristics of Cy3 labeled α-syn monomers (A), HNE oligomers (B) and fibrils (C). Scale bars = 500 nm. (PDF) [file pone.0168700.s001.pdf]

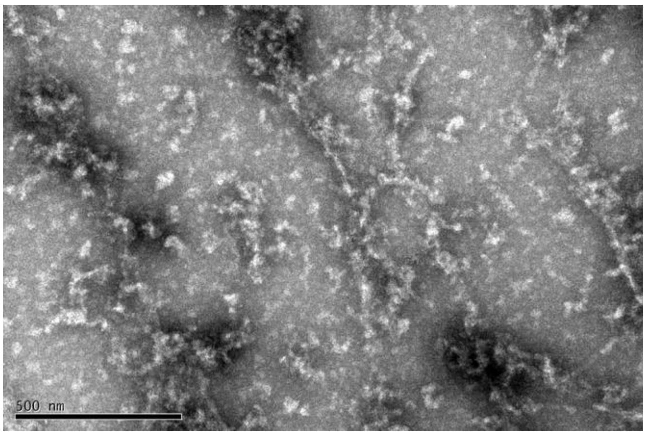

Monomers

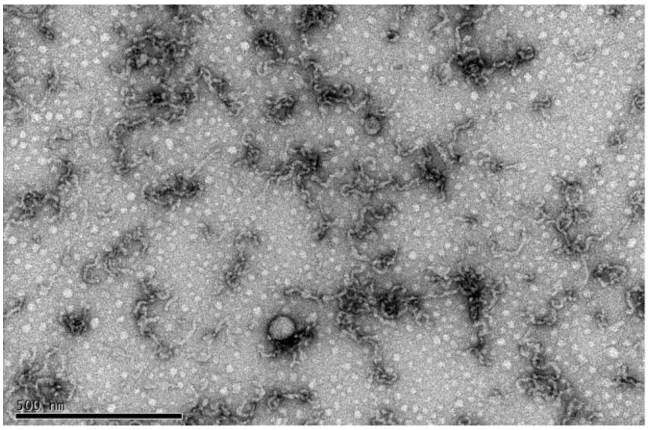

HNE oligomers

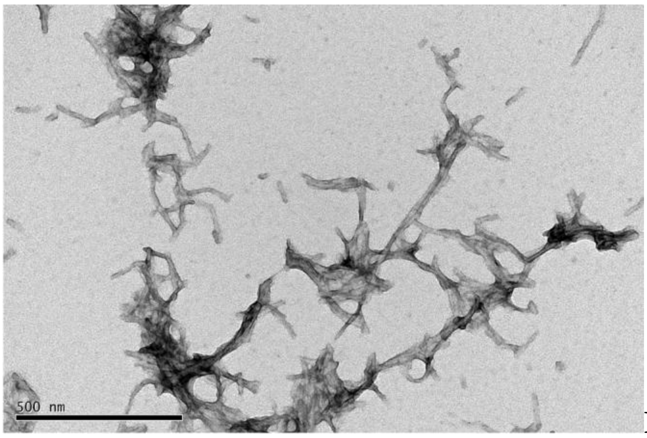

Fibrills

S1 Fig. Electron microscopy characterization of labeled α-syn species Standard transmission electron microscopy (TEM) images show the different, and typical, characteristics of Cy3 labeled α-syn monomers (A), HNE oligomers (B) and fibrils (C). Scale bars = 500 nm.
