# Supplementary material for: Aggregated Alpha-Synuclein Transfer Efficiently between Cultured Human Neuron-Like Cells and Localize to Lysosomes
Source: PLoS One. 2016 Dec 28;11(12):e0168700. doi: 10.1371/journal.pone.0168700 (PMC5193351; doi:10.1371/journal.pone.0168700)
Supplement: S2 Fig — (A) XTT assay was performed on donor cells according to the manufacturer’s instructions 24h after incubation with each Cy3 labeled α-syn species. Only monomeric alpha-synuclein showed a significant decrease in cell viability after 24h. n = 3. Data are presented as mean ± SEM, ANOVA with Bonferroni’s correction. (B) As a further measure of early cellular toxicity, beading of GFP labeled tubulin was examined as described previously [1,2]. 7 days after addition of 1μM Cy3 labeled α-syn (red), no indication of tubulin beading could be seen in response to any of the α-syn species. Blue = DAPI. Images are representative, n = 3. (PDF) [file pone.0168700.s002.pdf]

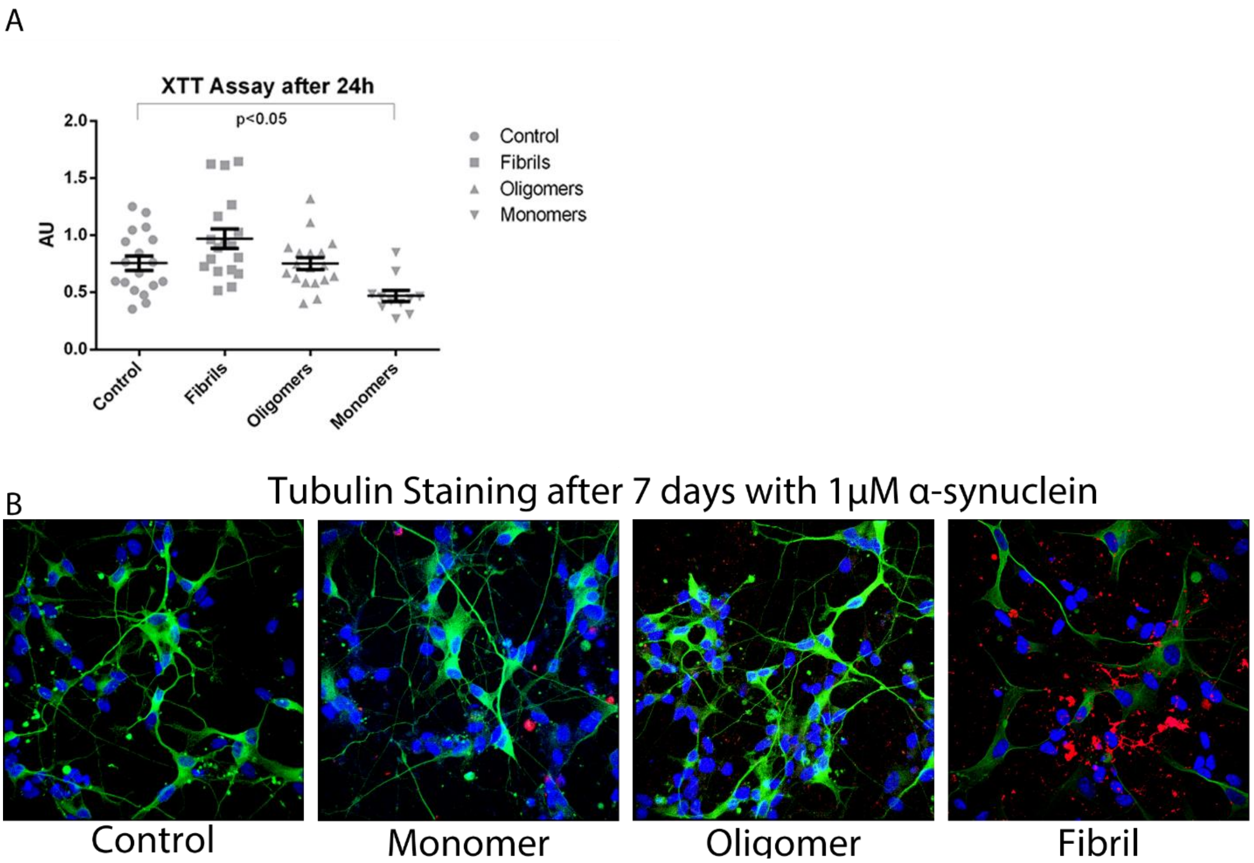

S2 Fig. Toxicity of alpha-synuclein. **(A)** XTT assay was performed on donor cells according to the manufacturer's instructions 24h after incubation with each Cy3 labeled α-syn species. Only monomeric alpha-synuclein showed a significant decrease in cell viability after 24h. n=3. Data are presented as mean ± SEM, ANOVA with Bonferroni's correction. **(B)** As a further measure of early cellular toxicity, beading of GFP labeled tubulin was examined as described previously [1,2]. 7 days after addition of 1µM Cy3 labeled α-syn (red), no indication of tubulin beading could be seen in response to any of the α-syn species. Blue=DAPI. Images are representative, n=3.

References

1. Nath S, Agholme L, Kurudenkandy F, Granseth B, Marcusson J, Hallbeck M. Spreading of neurodegenerative pathology via neuron-to-neuron transmission of β-amyloid. The Journal of neuroscience : the official journal of the Society for Neuroscience. 2012;32(26):8767-77. doi: 10.1523/JNEUROSCI.0615-12.2012.
2. Agholme L, Nath S, Domert J, Marcusson J, Kagedal K, Hallbeck M. Proteasome inhibition induces stress kinase dependent transport deficits - Implications for Alzheimer's disease. Molecular and cellular neurosciences. 2013. doi: 10.1016/j.mcn.2013.11.001
