# Supplementary material for: Aggregated Alpha-Synuclein Transfer Efficiently between Cultured Human Neuron-Like Cells and Localize to Lysosomes
Source: PLoS One. 2016 Dec 28;11(12):e0168700. doi: 10.1371/journal.pone.0168700 (PMC5193351; doi:10.1371/journal.pone.0168700)
Supplement: S3 Fig — The supernatant from donor cells was collected 24h after the same uptake conditions as in all other experiments. Extracellular Cy3 fluorescence was significantly increased in fibril treated cells relative to oligomer and monomer treated cells supporting an increased release of fibrils versus monomers or oligomers. n = 3 for each group. Data are presented as mean ± SEM, ANOVA with Bonferroni’s correction. (PDF) [file pone.0168700.s003.pdf]

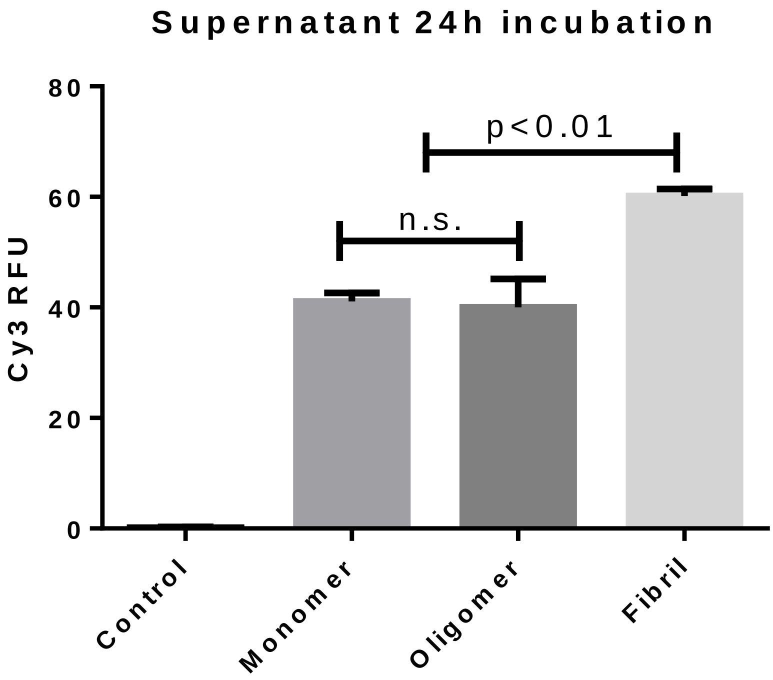

S3 Fig. Extracellular secretion from donor cells. The supernatant from donor cells was collected 24h after the same uptake conditions as in all other experiments. Extracellular Cy3 fluorescence was significantly increased in fibril treated cells relative to oligomer and monomer treated cells supporting an increased release of fibrils versus monomers or oligomers. n=3 for each group. Data are presented as mean ± SEM, ANOVA with Bonferroni’s correction.
